# Timing Intervals Using Population Synchrony and Spike Timing Dependent Plasticity

**DOI:** 10.3389/fncom.2016.00123

**Published:** 2016-12-01

**Authors:** Wei Xu, Stuart N. Baker

**Affiliations:** Movement Laboratory, Institute of Neuroscience, Medical School, Newcastle UniversityNewcastle Upon Tyne, UK

**Keywords:** timing, synchrony, synaptic plasticity

## Abstract

We present a computational model by which ensembles of regularly spiking neurons can encode different time intervals through synchronous firing. We show that a neuron responding to a large population of convergent inputs has the potential to learn to produce an appropriately-timed output via spike-time dependent plasticity. We explain why temporal variability of this population synchrony increases with increasing time intervals. We also show that the scalar property of timing and its violation at short intervals can be explained by the spike-wise accumulation of jitter in the inter-spike intervals of timing neurons. We explore how the challenge of encoding longer time intervals can be overcome and conclude that this may involve a switch to a different population of neurons with lower firing rate, with the added effect of producing an earlier bias in response. Experimental data on human timing performance show features in agreement with the model's output.

## Introduction

Timing is an essential part of producing voluntary coordinated movements. Precisely timed sequences of muscular contractions are required to generate a range of behaviors from speech production to locomotion. These voluntary movements require neuronal timing systems that are both precise and flexible.

The neural mechanism of timing has been studied via a variety of experimental and theoretical techniques, and has implicated brain regions including the cerebellum, striatum, and neocortex (Ivry, 1997; Malapani et al., 1998a,b; Matell and Meck, 2000; Klein-Flügge et al., 2011). The prevailing theories of interval timing involve clock signals that are collated and/or interpreted in order to gauge the passage of time. Two major (and mutually non-exclusive) theories are the “pacemaker-accumulator model” and the “beat-frequency” model (Buhusi and Meck, 2005). Both require some form of oscillatory signal, which is found in abundance in the brain (Buzsáki and Draguhn, 2004).

Neurons generally communicate with each other and generate behavior via the relative timing of their action potentials. Any timing mechanism must emerge out of, and be limited by the mechanisms that modulate action potential timing. Neurons are temporally imprecise and therefore make poor clocks; besides, even good clocks can be subject to temporal inaccuracies. For example, in electronic engineering analog-to-digital conversion is subject to jitter in the sampling interval. Every effort is made to minimize sampling clock jitter but there is always a certain degree of sampling error that is in proportion to the duration of the recorded signal, which inevitably distorts the recorded data (Da Dalt et al., 2002). The temporal precision of neurons is orders of magnitude worse than that of electronic components, and hence trains of action potentials accumulate temporal jitter very quickly. Any neuronal clock that depends on repetitive action potentials becomes increasingly limited in temporal accuracy with the passage of time. Models of neuronal time-keeping should take this unavoidable biological limitation into account. Additionally, it has long been established that learning generally takes place via synaptic plasticity. Therefore, we wish to investigate the properties and limitations of a system that combines synaptic plasticity with noisy oscillators to encode different time intervals. To our knowledge previous models of interval timing have not explicitly accounted for the noisy nature of neurons or its effect on synaptic plasticity. Our model takes into account the accumulation of temporal jitter and shows that such populations of neurons can be used to encode intervals from hundreds of milliseconds to several seconds—times relevant for coordinated movements. We find that the neuronal noise limits the length of time intervals that can be encoded. Moreover we find that the generally-recognized rules of spike dependent synaptic plasticity cause a bias in the output for certain time intervals. The output of the model is compared with human subjects performing an interval timing task.

The cerebellum has been heavily implicated in controlling the timing of movements: many clinical manifestations of cerebellar lesions are failures of timing. For example patients with cerebellar damage generate mistimed agonist and antagonist muscle contractions resulting in jerky movements and dysmetria (Holmes, 1939). Here we present a model for timing intervals around 1 s, based on previous *in vivo* data from a pre-cerebellar nucleus that has the characteristics of a neuronal clock (Xu et al., 2013).

## Materials and methods

**Figure 1:**
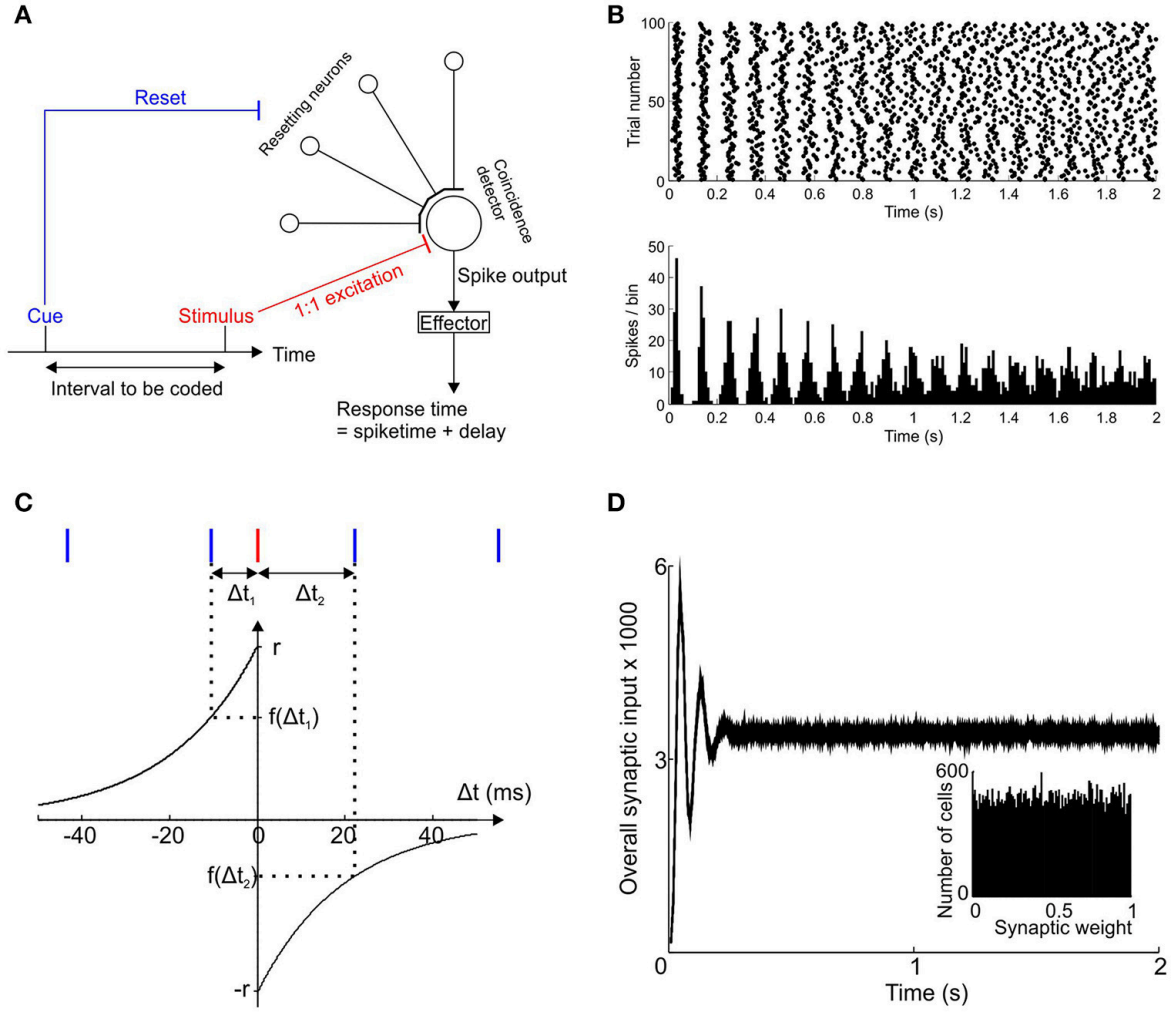
**Scheme of interval timing model. (A)** Schematic diagram showing the convergence of a bank of independent pacemakers onto a coincidence detector neuron. The cue signifying the start of the time interval resets all pacemakers and the stimulus signifying the end of the interval causes the coincidence detector neuron to fire. **(B)** Post-stimulus time histogram and raster for a single example pacemaker neuron after phase reset at *t* = 0 (100 trials). **(C)** Spike time dependent plasticity function of the post-synaptic neuron used to update the synaptic weights of every pacemaker. Pacemaker spikes (blue) that occur before the stimulus-driven spike (red) cause potentiation of the synaptic weight; pacemaker spikes after the stimulus-driven spike reduce the synaptic weight. **(D)** Synaptic input to the coincidence detector neuron, averaged over 100 trials with a learning rate of zero and target time provisionally set at 0.5 s, using a population of 50,000 pacemaker cells. Inset shows distribution of synaptic weights at trial 100.

Our model of interval timing consists of a large bank of independently and regularly spiking pacemaker neurons that converge onto a coincidence detector (Figure 1A). The time interval to be encoded is demarcated at its start by a sensory cue that resets the oscillatory phase of all the pacemakers and at its end by another sensory stimulus that invariably causes the coincidence detector to fire (stimulus-evoked spike).

Each pacemaker neuron sends an excitatory synapse onto a coincidence detector neuron to produce an excitatory post-synaptic potential (EPSP). If enough EPSPs are received within a 10 ms time window (similar order to neuronal membrane time constants—McCormick et al., 1985) then a threshold is exceeded and the post-synaptic coincidence detector fires a spike.

### Properties of pacemakers

The pacemaker neurons emit pulses (spikes) which accumulate temporal jitter in accordance with the rule demonstrated in the lateral reticular nucleus (LRN) neurons of the rat (Xu et al., 2013; experiments and modeling carried out by current first author) and restated here:

(1) $$\begin{array}{l} {\text{S}}_{\text{n}}=\left(\bar{{\text{S}}_{1}}+{\text{J}}_{\text{First}}\right)+(\text{n}-1)\text{I}+\sum_{\text{k}=1}^{\text{n}-1}{\text{J}}_{\text{Interval}\;\text{k}}. \end{array}$$

S_n_ is the time of the n'th spike in a spike train after phase reset, $\bar{{\text{S}}_{1}}$ is the expected time of the first post-reset spike, J_First_ is a random variable for the temporal jitter of the first spike, I is the expected value of interspike interval and J_Interval k_ is a random variable for the temporal jitter in the k'th interspike interval.

All simulations described in this paper used a population of 50,000 pacemaker neurons unless otherwise stated. For each simulated neuron the value of $\bar{{\text{S}}_{1}}$ and I were chosen randomly from Gaussian distributions whose means and standard deviations were taken from experimental data in rat LRN (Xu et al., 2013). The means were 48.6 and 76.7 ms and standard deviations 11.9 and 6.2 ms for $\bar{{\text{S}}_{1}}$ and I respectively. For a given cell and trial, J_First_ was chosen from a zero-mean Gaussian distribution with standard deviation $\text{C}{\text{V}}_{\text{First}}\;\bar{{\text{S}}_{1}}$, where CV_First_ is the coefficient of variation of the times of the first post-reset spike. Similarly, the random variable J_Interval k_ was taken from a zero-mean Gaussian distribution with standard deviation of CV_Interval_ I. Jitter values could therefore be negative or positive; the temporal variability of both the first post-reset spike and the subsequent interspike intervals were scaled according to their mean values. Both coefficients of variation were determined based on experimental recordings (CV_First_ = 0.245; CV_Interval_ = 0.08). An example post-reset time histogram for a simulated pacemaker cells is shown in Figure 1B.

### Spike time dependent plasticity of convergent pacemaker inputs

The general spike-time-dependent-plasticity (STDP) rule followed here is that synapses that were active just before post-synaptic firing were potentiated, whereas those active just after post-synaptic firing were depressed. STDP of the synapses between pacemakers and the coincidence detector neuron was determined by a pair of exponential functions as shown in Figure 1C. This function was taken from Song et al. (2000), based on previous experimental work (Markram et al., 1997; Bi and Poo, 1998; Debanne et al., 1998). The time constant τ of the exponentials was 20 ms. The time interval of the pre-synaptic pacemaker spikes immediately before and after the stimulus-driven spike are respectively denoted Δt_1_ and Δt_2_ (Δt_1_ < 0, Δt_2_ > 0). The overall sign and magnitude of synaptic weight change after each trial was determined by the value of F where:

(2) $$\begin{array}{l} \text{F}=\text{f}\left(\Delta {\text{t}}_{1}\right)+f\left(\Delta {\text{t}}_{2}\right)\\ =\text{r exp}\left(\Delta {\text{t}}_{1}/\tau \right)-\text{r exp}\left(-\Delta {\text{t}}_{2}/\tau \right). \end{array}$$

The term r is the “learning rate” which determined the maximum possible amount of synaptic potentiation and depression per trial.

After each trial the value of F was then used to update the synaptic weight using a multiplicative rule (Rubin et al., 2001).

(3) $$\begin{array}{l} {\text{W}}_{\text{n}+1}={\text{W}}_{\text{n}}\;+\text{ ΔW},\\ \text{where ΔW}=\left(1-{\text{W}}_{\text{n}}\right)\text{F if F}>0\\ \text{and ΔW}=\left({\text{W}}_{\text{n}}\right)\text{F if F}<\;0. \end{array}$$

The current weight of a given synapse on the n'th trial, W_n_, was altered by the amount ΔW to give the new weight of the next trial, W_n+1_. The magnitude and sign of ΔW depend on how far W_n_ is currently from the upper or lower hard boundaries of the synaptic weights (respectively 1 and 0) and also on the magnitude and sign of F. If, on a given trial, the synaptic weight exceeded the maximal or minimal weight hard boundaries then it was set to equal that boundary. The naïve weights of synapses (at the start of trial 1) were randomly assigned from a uniform distribution between 0 and 1.

The post-synaptic response was derived for each trial by simulating a train of reset spikes for all pacemakers, binning the pacemaker spikes in 10 ms-wide bins (same order as neuronal membrane time constants; the onset of the first bin was at 0 ms, the time of the cue), multiplying the height of each bin by the synaptic weight for that cell and then summing across the population of pacemaker neurons.

### Post-synaptic threshold

According to the scheme proposed in Figure 1 the coincidence detector neuron can fire a spike either through being driven by sufficient population synchrony in input pacemakers, or by direct input from the stimulus that demarcates the end of the time interval. We model the coincidence detector neuron to fire first when its synaptic inputs first exceed its firing threshold—in reality the neuron may fire a train of spikes for the duration over which its synaptic inputs remain above threshold. We assume that the first output spike carries the most accurate timing information. If the pacemaker population synchrony is insufficient to make the coincidence detector fire then it would be made to fire a little later by the stimulus. In our scheme the first post-cue spike fired by the coincidence detector is used to encode the target time and this spike is then used to generate a response which necessarily occurs after an effector delay. This means that on trials where the pacemaker population synchrony is not enough to drive a spike (this depends both on the magnitude of synchrony and on the post-synaptic threshold), a response will always occur later than the stimulus that evoked it.

The post-synaptic threshold in the coincidence detector neuron was adjusted to minimize the total error of the response times.

Total error was defined as:

(4) $$\begin{array}{l} \text{E}=\sqrt{\frac{1}{50}\sum_{\text{n}=51}^{100}{\left({\text{r}}_{\text{n}}-\text{T}\right)}^{2}}\\ \;=\;\sqrt{\sigma_{\text{r}}^{2}+{\text{B}}^{2}}, \end{array}$$

where $\sigma_{r}^{2}$ denotes the variance of response times, and

(5) $$\begin{array}{l} {\text{r}}_{n}=\;{\text{t}}_{n}+\text{d} \end{array}$$

(6) $$\begin{array}{l} \text{B}=\bar{{\text{r}}_{\text{n}}}-\text{T}. \end{array}$$

r_n_ is the response time for the n'th trial ($\bar{{\text{r}}_{\text{n}}}$ is its mean over the last 50 trials) and T is the target time; t_n_ denotes the time of the earliest post-reset spike of the coincidence detector neuron and d denotes the effector delay. The response variance and its mean bias ($\sigma_{r}^{2}$ and B) respectively denote the variance and mean bias of responses from trials 51 to 100. The value of E was calculated from the last 50 trials of the simulation, testing thresholds ranging from 1 to 30 standard deviations above baseline population response. The threshold that gave the lowest value of E was taken as the steady state post-synaptic threshold and its corresponding response times were taken as the learned response time.

### Psychophysics experiment

Twenty healthy human subjects (13 males and 7 females, aged between 20 and 30 except for one in his 40 s) were tested in an interval learning experiment in which a somatosensory stimulus denoted the interval start and a light flash denoted its end. Median nerve stimulation (side randomly selected) was used as the cue to denote the start of the time interval to be learned. Monophasic current pulses (0.1 ms duration) were delivered through pre-gelled silver/silver chloride electrodes attached to the volar aspect of the wrist using a Digitimer DS7A stimulator. The anode was applied over the carpal tunnel and the cathode was applied more proximally on the wrist. Current intensity was adjusted for each subject to be just below motor threshold (gauged by the twitching of thenar muscles). A 100 ms-long red LED flash at subject eye-level was used to signal the end of the interval to be learnt. Using the same hand being stimulated the subject operated a button with the thumb. He/she was instructed to try to press the button at the same time as the LED flash onset. Time intervals ranged from 0.3 to 2 s in 0.1 s steps. Each value of time interval was tested with 100 trials with a random inter-trial interval ranging between 2 and 3 s. Each subject was tested using a randomly selected subset of time intervals. These were picked from the whole set of intervals without replacement. Each time interval was tested with at least 3 subjects. Performance feedback was provided to the subject immediately after each trial in the form of a cursor on a computer screen whose horizontal position relative to the screen center (marked by a vertical line) represented the relative time of subject's button press to the onset of the LED flash (left indicated button press occurred before light flash, and right after). We assumed that this feedback error signal would allow adjustment of response threshold, which is a key component of our model. The subject was instructed to try to superimpose the cursor onto the center of the screen. The subjects' performance was analyzed using both the mean temporal error and timing variance of the button press. All experiments with human subjects were approved by the local ethics committee of Newcastle University's Faculty of Medical Sciences; subjects provided written informed consent to participate.

## Results

### Pacemakers accumulate spike time variance linearly

**Figure 2:**
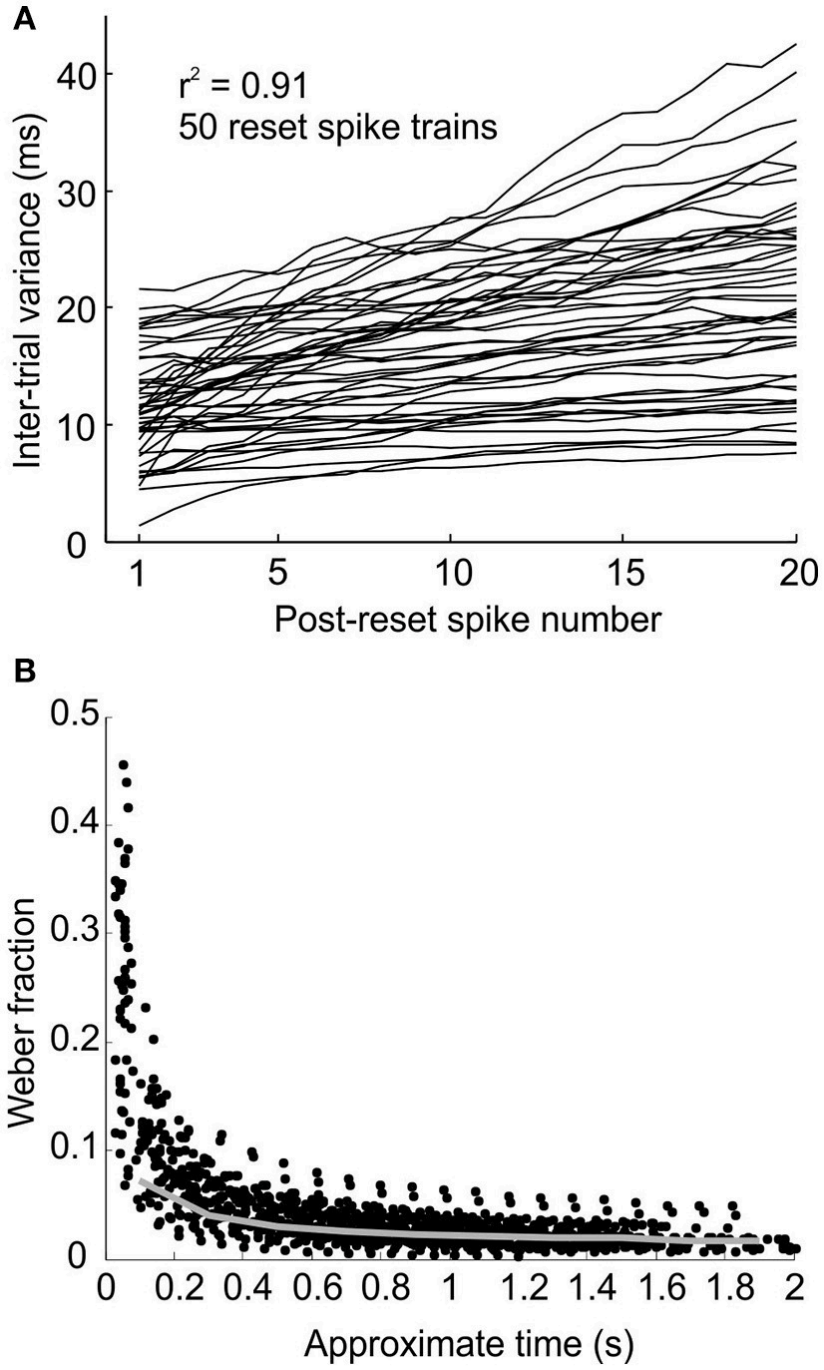
**Pacemakers accumulate temporal variance linearly with each subsequent spike. (A)** Plot of inter-trial variance for the n'th post-reset spike against post-reset spike number for 50 simulated reset spike trains. **(B)** Plot of Weber's fraction against estimate of post-reset time for simulated spikes in **(A)**. Gray line represents the mean binned Weber fraction calculated from the linear regression coefficients of the lines in **(A)** (represented by c in Equation 8).

The model assumes that repetitive pacemaker pulses are regenerated via intrinsic processes which have their own variability (supported by the *in vitro* data of Xu et al., 2013). Therefore, each pulse is associated with a degree of temporal jitter causing the times of its subsequent pulses to become increasingly uncertain. This is shown by the gradual smearing out of the peaks and troughs in the sample simulated post-reset time histogram in Figure 1B (100 trials). Unlike perfect oscillators, synchrony between two units with inter-spike intervals that are, on average, integer multiples of each other cannot occur with absolute periodicity or predictability, because of the accumulation of variability in successive intervals. One of the properties of the simulated pacemaker neurons is that the inter-trial variance of post-reset spikes ($\sigma_{\text{n}}^{2}$) accumulates in an approximately linear fashion with n because the variance of independent random variables (here inter-spike intervals) is additive (see Figure 2A, *r*^2^ = 0.91 ± 0.15 for 50 linear regressions—each line represents a different simulated post-reset spike train). If post-reset spike number, n, is taken as an approximation of time, then the Weber's fraction, defined as:

(7) $$\begin{array}{l} \text{K}=\sigma_{\text{n}}/\text{T}, \end{array}$$

where T$\;\sim \;\bar{{\text{S}}_{1}}$ + (n - 1)I, ($\bar{{\text{S}}_{1}}$ and I defined in equation 1 in Methods) takes on the shape as shown in Figure 2B. This is because linear accumulation of variance implies: $\sigma_{r}^{2}=$ cT Where c is the slope constant.

Substitution then gives:

(8) $$\begin{array}{l} \text{K}=\sqrt{\text{c}/\text{T}}. \end{array}$$

The plots of Weber Fraction vs. T from the model (Figure 2B) appear to follow this relationship.

### Synaptic input from pacemaker neurons

A large enough population of pacemakers will, by sheer chance, contain subpopulations that have increased spike synchrony with each other at certain times. This synchrony will not initially be apparent because their relative synaptic input strengths are not yet high enough. Synchrony between certain different pacemakers was used by the coincidence detector neuron in the model to encode time intervals. The learning strategy was to potentiate those synapses that happened to have been consistently active just before the coincidence detector neuron fired, eventually leading to those synapses being strong enough to drive the coincidence detector neuron in advance of the stimulus. For a given target time most of the population will not actually fire at the required time, therefore their inputs are not strengthened (and may in fact be weakened).

Such a system of population synchrony coding is constrained by the accumulation of temporal jitter in the pacemakers. Firstly the population of pacemakers required to encode an interval “sufficiently” will be larger than when using perfect oscillators. Secondly the performance of synchrony coding decreases with increasing time intervals, therefore the interval that can be encoded cannot be made arbitrarily long.

An example of the pre-synaptic input to the coincidence neuron for 100 trials without learning (i.e., learning rate *r* = 0) is shown in Figure 1D. The notional target time in this case was 0.5 s. An initial synchronization transient was followed by almost constant input to the coincidence detector neuron. Such a synchronization transient has previously been suggested to underlie the “event-related potentials” seen in EEG signals (Matell and Meck, 2000). The histogram in the inset to Figure 1D shows a uniform distribution of synaptic weights which, given the zero learning rate, was identical for all trials from 1 to 100. Clearly in the absence of learning the naïve system response was of no use for encoding the required time interval of 0.5 s.

**Figure 3:**
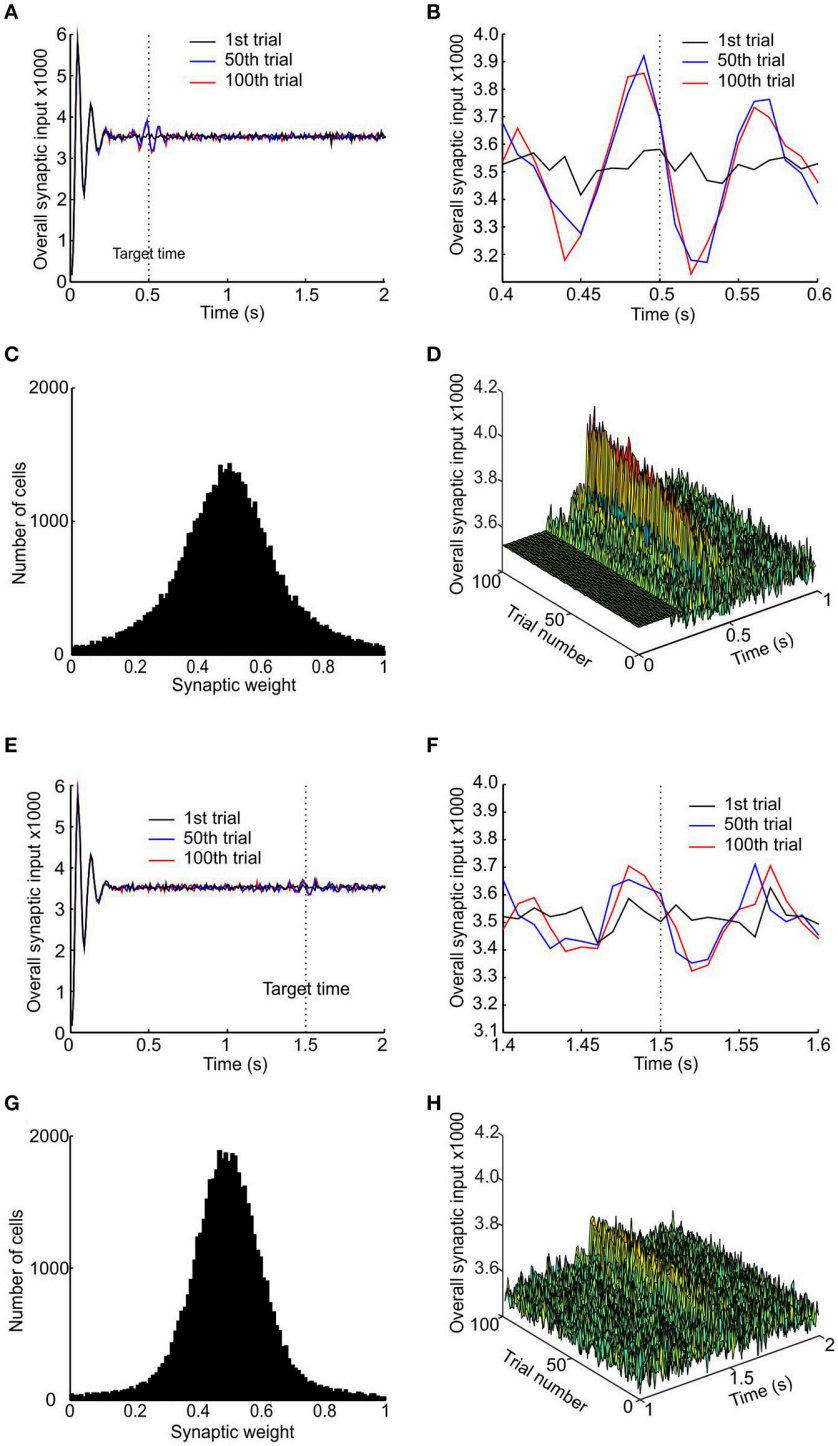
**Development of a peak in synaptic input to coincidence detector. (A)** Total synaptic input to the coincidence detector cell after the 1st, 50th, and 100th trial (respectively black, blue, and red traces) for target time of 0.5 s. **(B)** Enlarged trace of total synaptic input showing a peak developing slightly before the target time. **(C)** Distribution of synaptic weight after 100 trials. **(D)** Stacked plots of total synaptic input from trial 1 to 100, showing the development of the peak in the first 50 trials. **(E–H)** Same plots as **(A–D)** but using a target time of 1 s.

Figure 3 shows the results obtained with a learning rate *r* = 0.1, for two target times of 0.5 and 1.5 s. The pre-synaptic pacemaker inputs developed a local peak around the target time, which was present after 50 trials and remained unchanged at trial 100 (Figures 3A,E, shown on an expanded time scale in Figures 3B,F). For the target time of 0.5 s, the peak synaptic input clearly preceded the target time (Figure 3B); this was less clear for the 1.5 s target, where peaks before and after the target were of similar amplitude (Figure 3F). In both simulations, the synaptic weight distributions changed to become unimodal, with a peak around the synaptic weight of 0.5 at the end of the stimulation of 100 trials (Figures 3C,G). The increased synaptic input that develops over a number of trials is not due to increased temporal synchrony, but due to synchrony that already exists within the subpopulation being made more apparent in the input to the coincidence detector neuron by STDP.

One problem with any scheme which attempts a straightforward conversion of coincident synaptic inputs into response is that the initial synchronization transient immediately after population reset was much bigger than any subsequent response peaks. Only by suppressing this peak can a valid response be generated. Here, we achieved this by the simple but arbitrary expedient of setting the synaptic input in the first 250 ms after reset to equal the baseline mean (visible in Figure 3D as a flat band). A more biologically plausible mechanism to achieve the same effect might be a transient inhibition of the coincidence detector neuron.

**Figure 4:**
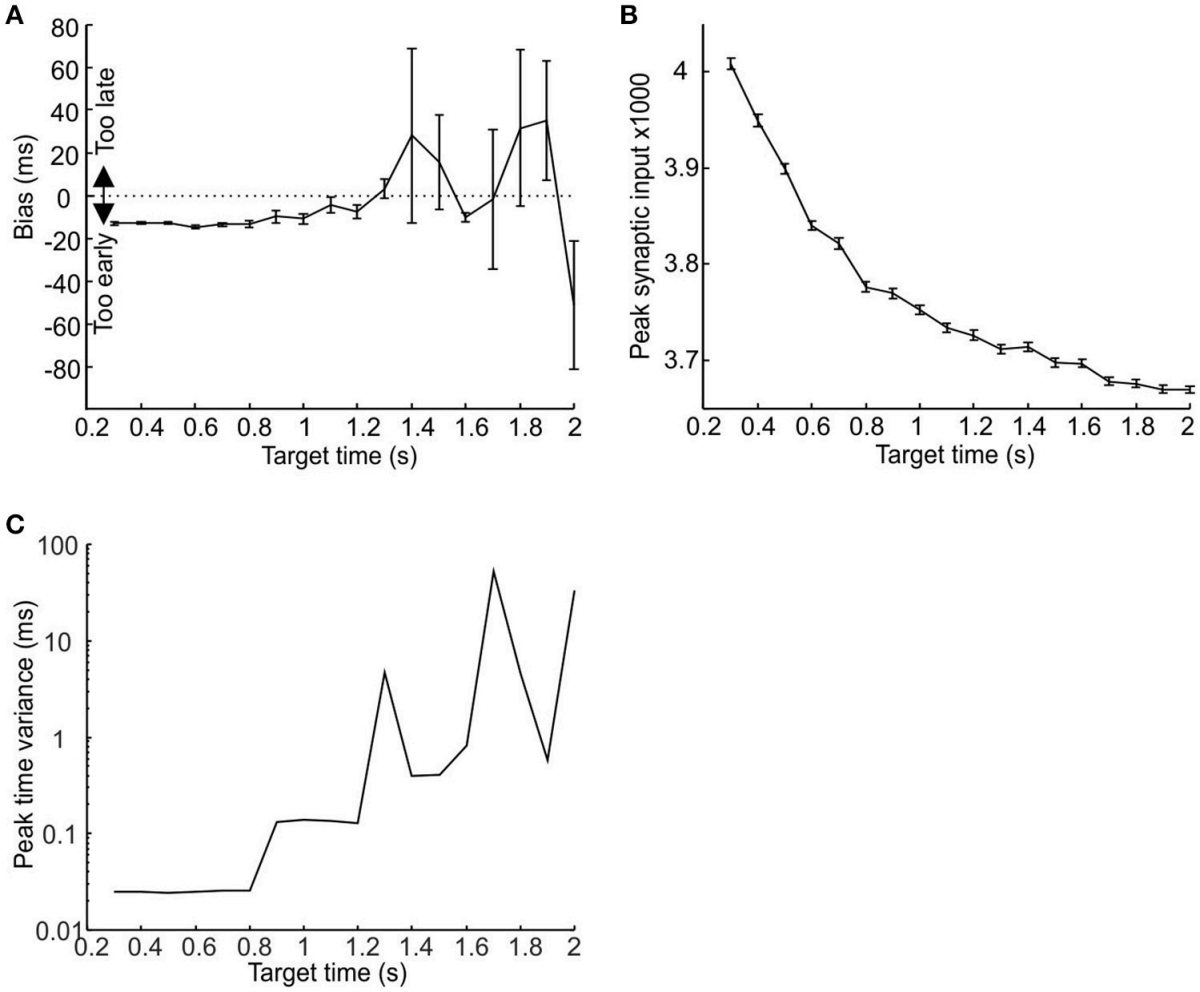
**The relationship between population response, target time and post-synaptic threshold. (A,B)** Plot of respectively synaptic input peak time and peak height against target time. **(C)** Semilog plot of peak time variance against target time. Measures have been made over trials 51–100; error bars denote standard error of the mean.

Given the odd symmetry of the STDP function the peaks in synaptic input for short target times (<1 s) tended to occur slightly but consistently earlier than target time, whereas for longer target times the results were more variable. Results for a single run of the model for target times from 0.3 to 2 s are shown in Figure 4A. Due to the accumulation of jitter with time it became increasingly unlikely for consistent synchrony to occur between different pacemakers with increasing time. Even after learning, synaptic input peaks were smaller for longer intervals (Figure 4B), with a corresponding rise in the trial-to-trial variance of peak time (Figure 4C).

The above results demonstrate that a large selection of pacemakers with randomly distributed periodicities and variabilities can be taught to produce a population response around the end of a time interval via spike time dependent plasticity. Moreover for shorter target time intervals the population response occurs with a slightly early bias. This is likely to be advantageous, since it may allow the system to compensate for a conduction delay in the generation of the response.

### Finding the optimal post-synaptic threshold

**Figure 5:**
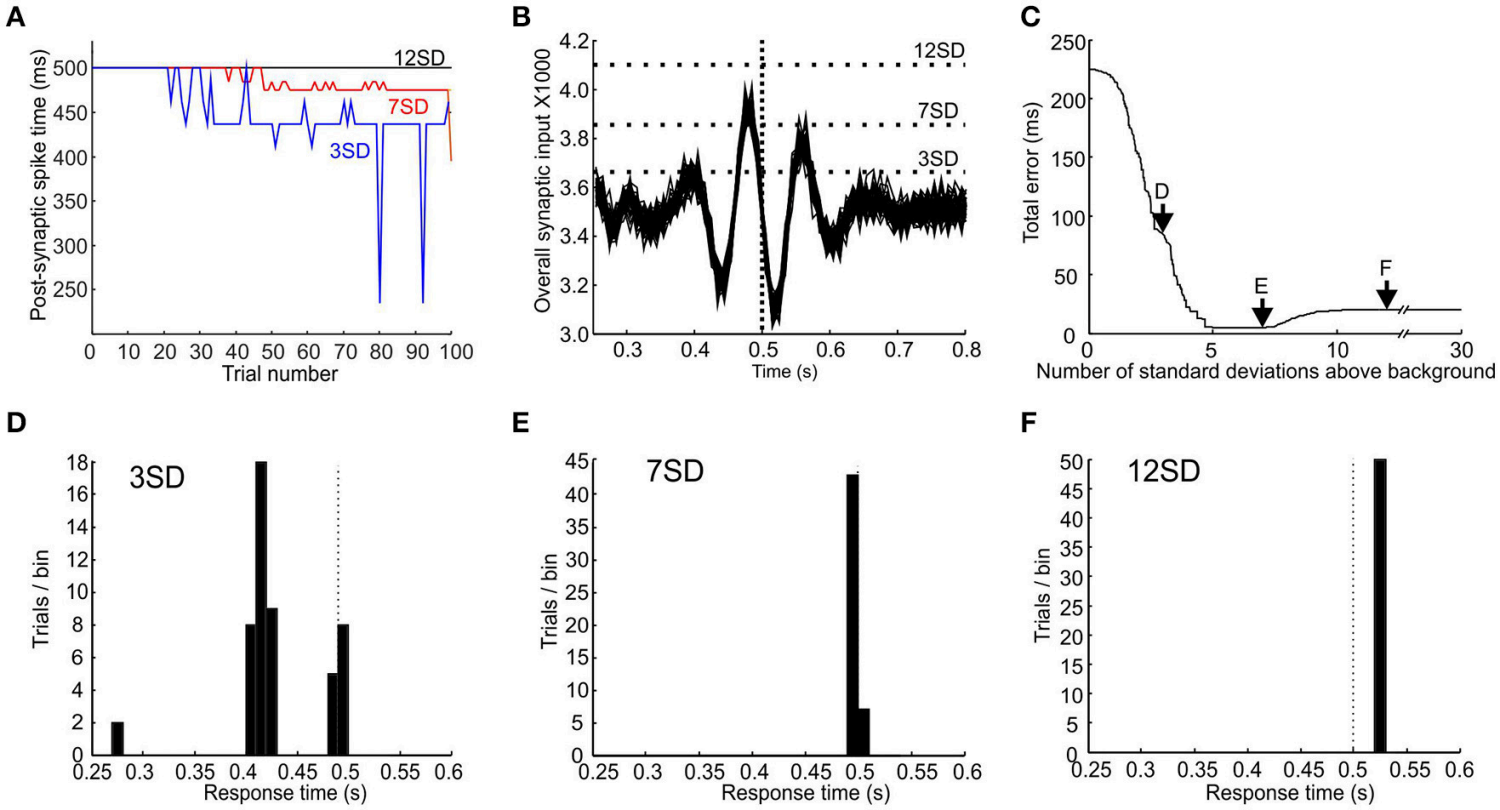
**Different thresholds give different response times and errors. (A)** Plot of the time at which synaptic input first crosses a post-synaptic threshold of 3 (blue), 7 (red), and 12 (black) standard deviations above baseline against trial number, for a target time of 0.5 s. **(B)** Plot of synaptic input from trials 51 to 100 for a target time of 0.5 s (indicated by vertical dotted line). Horizontal dotted lines indicate post-synaptic thresholds that are 3, 7, and 12 standard deviations about mean background response level. **(C)** Plot of total error vs. threshold level after an effector delay of 20 ms is added. Arrows mark thresholds levels of 3, 7, and 12 standard deviations above background. **(D–F)**, response-time distributions for threshold of 3, 7, and 12 standard deviations above background.

The spike time of the coincidence detector neuron depends not only on the synaptic weights of the inputs, but also on the firing threshold of the post-synaptic neuron. Figure 5A (blue line) illustrates the post-synaptic spiking times when we set the threshold simply to be 3 standard deviations above the baseline synaptic input established with the learning rate *r* = 0.1. Initially the output was stimulus-driven at the target time (here 0.5 s); as learning took place the spike output shifted earlier marking a response to pacemaker synchrony.

It is apparent from Figure 3 that the same post-synaptic threshold value is not necessarily optimal for all time intervals. Given that consistent coincident spiking becomes increasingly unlikely with time, a threshold value that produces an appropriately timed response for a short interval will be too high for a longer interval. The optimal threshold must be a trade-off. Too low a threshold will tend to respond to noise fluctuations, yielding a response that is too early and too temporally variable. By contrast, too high a threshold will miss a synaptic peak input generated by pacemaker synchrony, and produce a default response which is purely stimulus-driven. We envisage a scheme whereby the post-synaptic threshold is somehow optimized during learning to minimize both bias and variability. This process could occur via feedback of the difference between actual response time and desired response time for a given trial, which would be used as an error signal to shift the threshold to improve performance. Unfortunately, we do not know the detailed biological process by which such threshold optimization could be achieved, and are therefore not in a position to simulate a biologically-realistic mechanism (unlike the situation for the synaptic weight modification). We have therefore taken a phenomenological approach. We took the population response of the last 50 trials (when learning of synaptic weights has reached steady state) and tested all threshold values from 1 to 30 standard deviations (SDs) above mean background response. The threshold was selected which gave the minimal total error in response time (see Materials and Methods for definitions).

Figures 5B–F illustrates this process. Figure 5B shows an overlay of the synaptic input to the coincidence detector neuron for the last 50 trials; the target time was 0.5 s (vertical dotted line). It can be seen that thresholds that were too low (e.g., +3 SD) tended to give responses that were both too early and too variable (see distribution of response times in Figure 5D; assumed effector delay of 20 ms). Thresholds that were too high missed the peak in synaptic input altogether (+12 SD) and resulted in a default stimulus-driven response that was too late due to effector delay (distribution in Figure 5F). Figure 5C plots the total error vs. the threshold value. An optimal threshold existed from 5 to 7.2 SDs above background, which effectively minimized the total error value in response times (distribution for threshold of 7 SD above background shown in Figure 5E).

### Finding optimal learning rate and realistic effector delay

**Figure 6:**
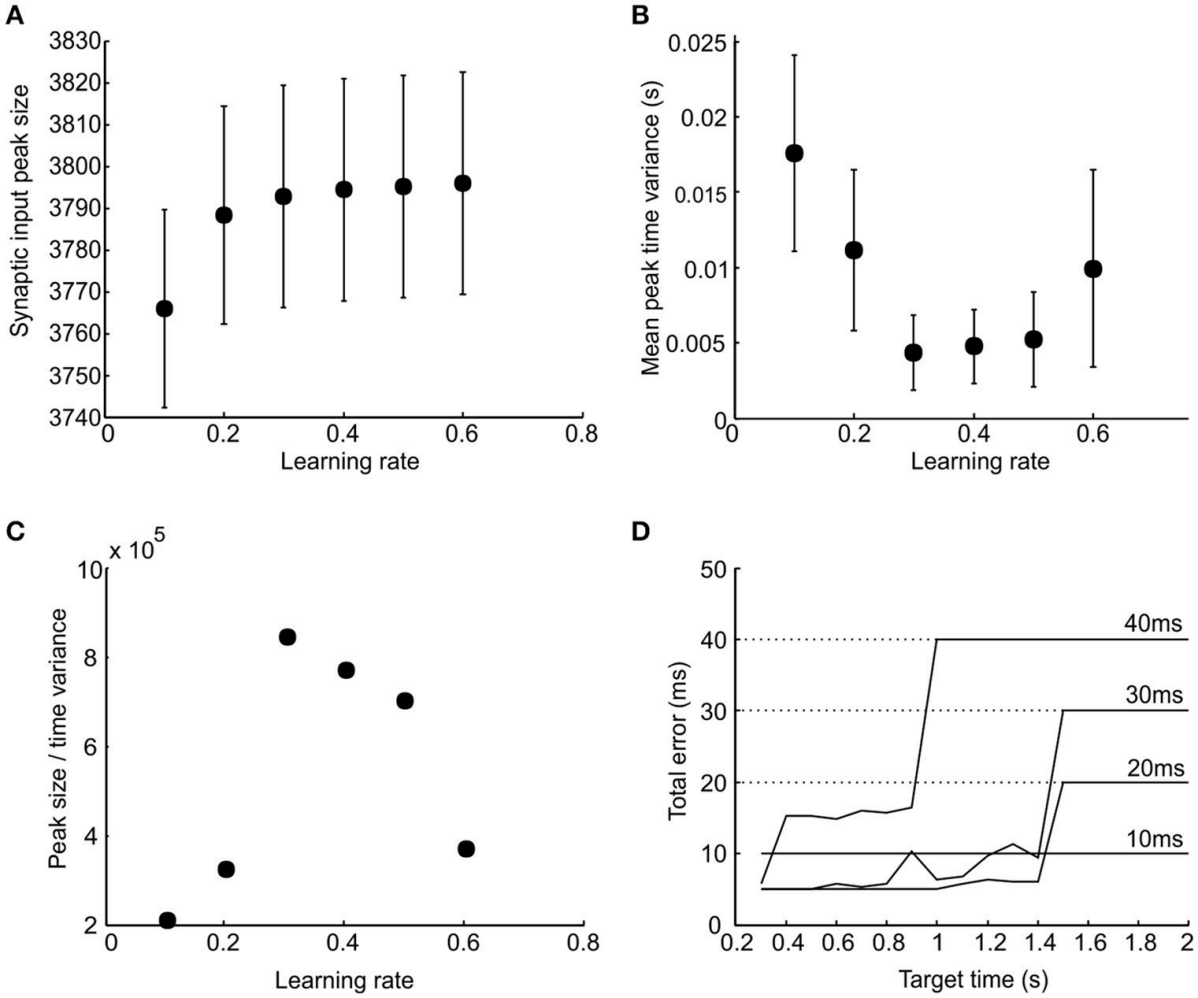
**Influence of learning rate and effector delay. (A,B)** Respective plots of total synaptic input peak amplitude and the variance in its time as a function of learning rate (error bars indicated SEM). **(C)** The ratio of the peak amplitude to peak time variance plotted against learning rate. **(D)** Total error in response time plotted against target time for four different values of effector delay (labeled); learning rate was fixed at 0.3. Dotted horizontal lines indicate the total errors produced by stimulus-evoked responses if there was no learning.

The ability of population synchrony reliably to encode a time interval depends both on its peak synaptic input value and the variability of the peak in time. A good learning performance would be one that generates both a peak input at the correct time and a low variance in the time of its peak (i.e., a good performance should be both accurate and consistent). To investigate the relationship between learning rate and performance and to find its optimal value, we plotted the peak synaptic input magnitude (Figure 6A) and its time variance (Figure 6B) against learning rate. The ratio of peak synchrony magnitude to time variance is shown in Figure 6C, which conveniently represents a compound performance measure. For all plots, results have been averaged across a range of target times from 0.3 to 2 s. There was a peak in performance around *r* = 0.3. It can be seen in Figure 6C that excessively high learning rates degrades performance. This is because it causes potentiation of chance coincident synapses, giving an excessively noisy population response. We therefore use a learning rate of *r* = 0.3 in subsequent simulations.

The effector delay has a subtle effect on the output of our model, which is examined in Figure 6D. This plots the total error vs. target time. In each case, the optimal value of firing threshold has been used. If the delay is set to zero, stimulus-driven spikes will occur at exactly the right time, with no jitter. There is thus no benefit in learning synchrony-driven responses. Even for small effector delays, learning conveys no benefit, and the best strategy is simply to respond to the stimulus—the small but consistent error at all target times (10 ms line in Figure 6D) is less than the random error which would be produced by attempting to learn the delay. For longer effector delays, the optimal performance was achieved by using the learned synchrony response for short target times, but switching to stimulus-driven responses for longer times. We used an effector delay of 20 ms to illustrate the detailed behavior further in the following section.

### Simulating response times for different population sizes

**Figure 7:**
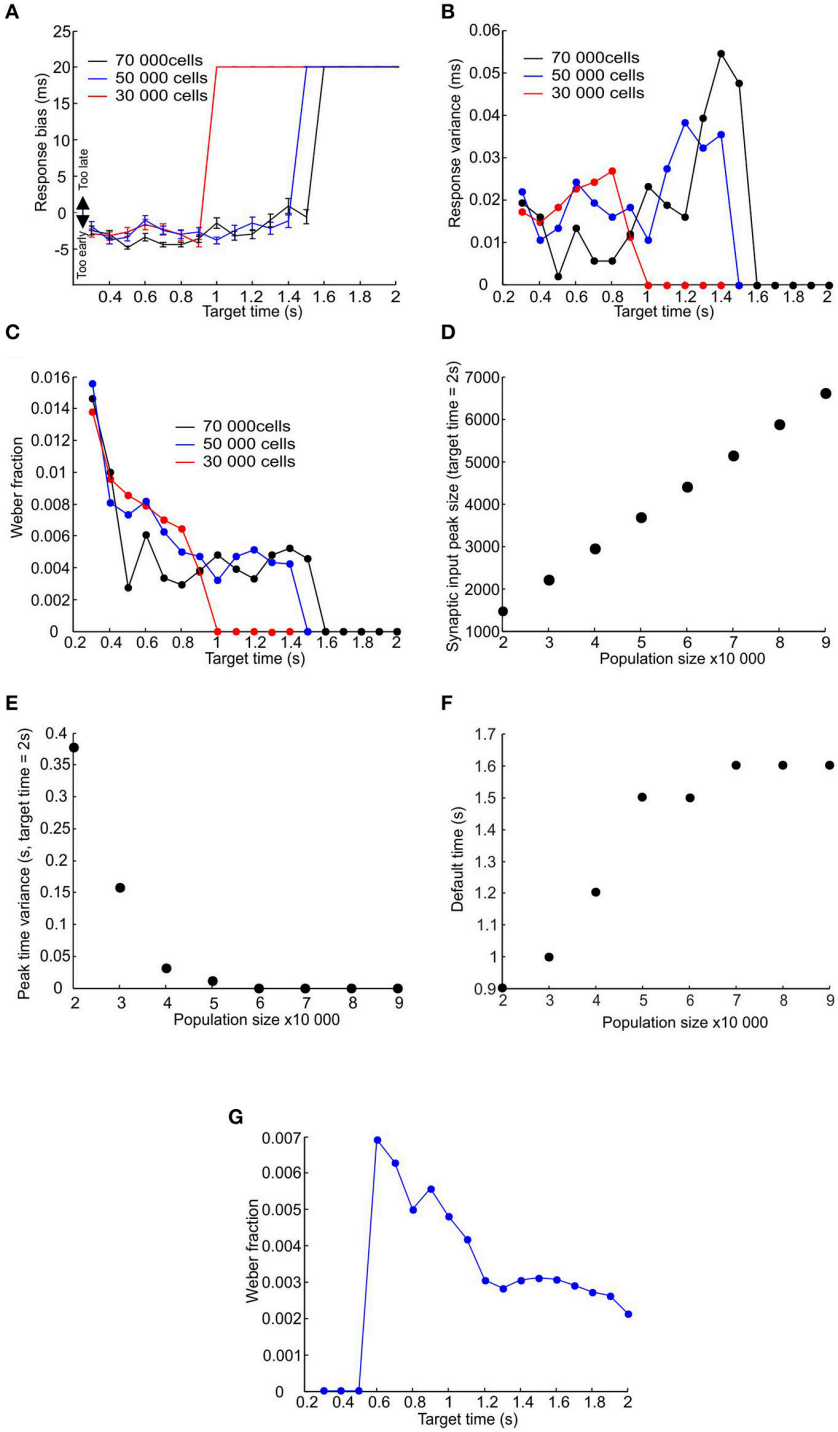
**Failure of learned responses for longer time intervals. (A–C)** Plot of response bias, response variance and Weber fraction against target time for three sizes of pacemaker population (30,000, 50,000, and 70,000 represented by red, blue and black traces). **(D–F)** Plot of mean amplitude of the peak in total synaptic input to the coincidence detector cell, the time variance of that peak, and the time at which the system defaults to the stimulus driven response against population size (measures have been calculated for a target time of 2 s; error bars are smaller than the size of the data points. **(G)** Plot of the Weber fraction against target time where the jitter of the first post-reset spike was set to zero. Population size 50,000.

Using a learning rate *r* = 0.3 (Figure 6C), an effector delay of 20 ms (Figure 6D) and a post-synaptic threshold chosen to minimize total error, we investigated the model performance in more detail for target times from 0.3 to 2 s. All measures were computed after learning had stabilized, using the last 50 trials of a 100 trial stimulation, and were tested with three different pacemaker population sizes (30,000, 50,000, and 70,000 units). Figure 7A shows the bias of response times, defined as the difference between the mean response and the target time (negative values represent a tendency to respond too early). For the smallest population examined, there was a slight but consistent bias toward responding early for target times shorter than 0.9 s (Figure 7A red trace); for longer intervals the system defaulted to responding with the stimulus-driven spike, representing the learned synchronization response's failure to produce an accurate and consistent enough response. This led to a late bias equal to the 20 ms effector delay. The learned response failed later for larger populations, but in all cases there was an eventual default to stimulus-driven reactions before target times reached 2 s (Figure 7A blue and black traces).

Figure 7B shows that the response time variance increased for longer target times before dropping down to zero for target times where the system has defaulted to the stimulus-driven response. The relatively constant variance (and therefore standard deviation) values for shorter target times resulted in the Weber fraction (σ/T) exhibiting an upward-going tail for shorter target times (Figure 7C—a similar relationship to Figure 2B). The ability to learn longer time intervals by larger populations is due to the randomly distributed nature of pacemaker parameters. This results in a larger number of pacemakers that are appropriate to within a small margin of error for a given time interval. Figures 7D,E show that for a given time interval (in this case 2 s) the amplitude of the synaptic input peak increased and its variance in time decreased with increasing population size. This is why larger populations can produce learned responses for longer time intervals (Figure 7F).

The relationship between the Weber fraction and target time has been previously attributed to the presence of a time-invariant source of variability that gains proportionately more weight for smaller time intervals (Getty, 1975). In our model the time-invariant source of variability is the jitter in the time of the first post-reset spike (the term J_First_ in Equation 1). However, even if we set this jitter value to zero some of the upward slope was still preserved (Figure 7F); as expected the effective encoding time is also extended. We suggest that the upward slope of the Weber fraction is due not only to the time-invariant jitter in the timing of the first post-reset spike, but also to the linear accumulation of jitter in subsequent spikes.

### Timing behavior may be limited by underlying neuronal network properties

The simulation results presented above suggest that if interval timing is fundamentally driven by resetting of convergent pacemakers whose impulses accumulate temporal variance linearly, then behavior should be subject to certain limitations imposed by such a strategy. One limitation would be that subjects cannot encode longer intervals as accurately as shorter intervals; and that beyond a certain duration the subject would completely fail to encode the interval (Figure 7B). Another limitation would be that the Weber's fraction of subjects' response increases as the time interval to be encoded decreases (Figure 7C). Naturally human performance may have many other limitations produced by the transformation of the low-level output represented by our model to an overt motor behavior. However, if processes similar to those which we have modeled are at work in human interval timing, it may be possible to discern their signature in psychophysical data.

**Figure 8:**
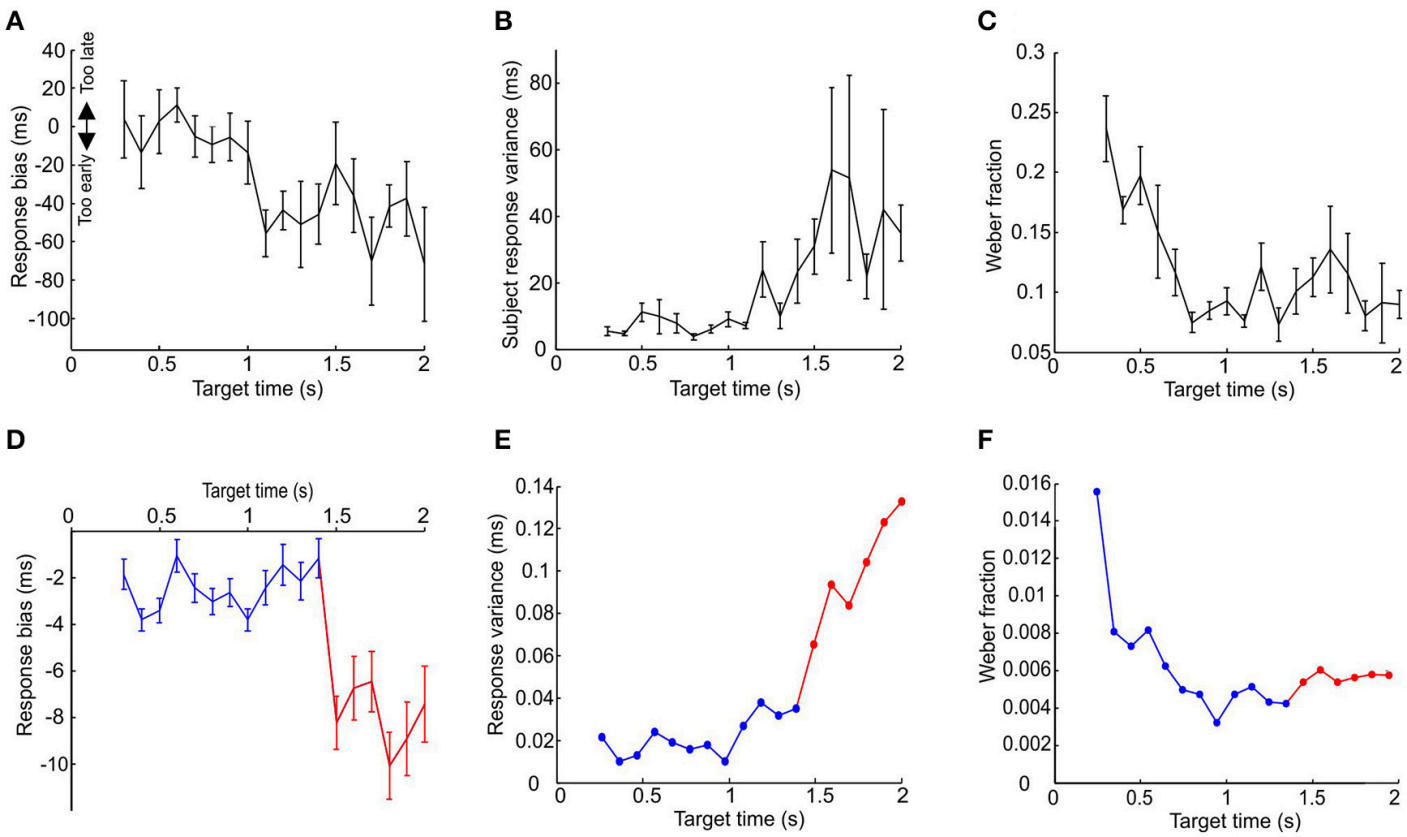
**Comparison between model and psychophysics data. (A–C)** Response variance, Weber fraction and bias plotted against target time for human subjects performing an interval timing task. Points are mean±SEM, and are calculated on the last 50 trials out of a total of 100 performed for each subject at each interval; between 3 and 10 subjects contributed to each data point. **(D–F)** Plot of response variance, Weber fraction and bias for simulated data. Blue traces are responses using a population of 50,000 pacemaker cells with properties as in all previous figures. Red traces illustrate the responses of the same sized population of pacemakers with interspike intervals double that used previously.

In order to examine this, we devised an interval learning experiment to compare the results of the above simulations with human performance. The cue demarcating the start of the interval was a median nerve stimulus and the target time was indicated by a light flash (see Materials and Methods). The variance, Weber fraction and response bias for the last 50 trials are plotted against the target time in Figures 8A–C. Subjects showed significantly earlier bias and greater response variance for longer target times and significantly higher value of Weber's fraction for smaller target times (all *P* < 0.05, one-way ANOVA).

For longer target times the human results did not seem to suffer the predicted limitation. The model predicts that the subjects will fail to learn the longer time intervals and default to responding by reacting to the stimulus, thereby giving a late bias and a very low response time jitter (see Figures 7A,B). However, in reality the subjects responded with an increasingly early bias and increasingly larger response time variance for target times over 1 s (*P* < 0.05, one-way ANOVA). This result is also at odds with the postulate that the effectiveness of learning is extended for longer intervals by recruiting more pacemakers. This is because recruiting more pacemakers will not lead to an increasingly earlier bias for longer time intervals (see Figure 7A).

One method the subjects might have used to overcome the predicted limitation is to use a separate and slower population of pacemakers for longer time intervals. A slower population of pacemakers will, on average, give an earlier response bias. The slight early bias shown by the model for shorter target times is due to the odd symmetry of the STDP function, resulting in the potentiation of synaptic inputs that fire slightly before the target time. If the average inter-spike interval of pacemakers is increased, then for a given spike pair which fall at random before and after the stimulus the values of both Δt_1_ and Δt_2_ (see Figure 2A) would be increased by on average the same amount, resulting in the same ratio of potentiation and depression (because potentiation and depression have the same time constant). However, if a spike occurs consistently just before the stimulus, the spike after the stimulus will be further away if the inter-spike interval is increased. Synaptic potentiation due to the preceding spike will thus be less canceled by depression by the succeeding spike. Population synchrony will then start to increase at an earlier time, and reach threshold earlier.

In order to illustrate how two populations of pacemakers might replicate the human data, we used the population of pacemakers from Figure 7 up to target times of 1.4 s, the point at which learning fails (for 50,000 units, blue traces in Figures 8D–F). For longer target times we used pacemaker cells which had double the average inter-spike interval of the original population (also 50,000 units, red traces in Figures 7D–F). The inter-spike interval jitter was scaled up proportionately; the optimal learning rate and minimal realistic effector delay was derived as above. It can be seen that the simulated responses of the slower population had a much earlier bias and the response variance continued to increase up to a target time of 2 s, similar to the experimental data.

## Discussion

We present here a variation of the beat-frequency coincidence detection model (Miall, 1989; Matell and Meck, 2000; Buhusi and Meck, 2005) using noisy pacemakers whose timing properties are derived from prior *in vivo* data (Xu et al., 2013). We show that a large population of resetting pacemakers with randomly distributed intrinsic timing parameters and millisecond timescales can be taught to encode specific time intervals in the seconds range using population synchrony and spike time dependent plasticity (Markram et al., 1997; Bi and Poo, 1998).

Previous models of interval timing tend to involve both pacemakers and an accumulator that keeps a running count of the number of pulses (Penney et al., 2000; Meck and Benson, 2002; Buhusi and Meck, 2005; Lustig and Meck, 2005). Our model does not require an accumulator. In this respect it is similar to the scheme of Miall (1989) in that the pacemakers that happen consistently to fire a pulse at the right time have their inputs potentiated via synaptic plasticity (and inappropriate pacemakers are suppressed) leading eventually to the selection of a useful subpopulation of inputs. However, our model also explains increasing variability in the responses encoding longer time intervals and the violation of the scalar property for short intervals.

### Physiological basis of model

We make no direct quantitative comparisons between our model and our psychophysics data and only use the human experiment to try to falsify the fundamental principles of the model. Although the actual numerical values of our model come from those measured in neurons of the LRN (Xu et al., 2013) the general timing principle of our model could apply to other systems in the brain such as the basal ganglia (Oprisan and Buhusi, 2011). The LRN is a major pre-cerebellar brainstem nucleus that projects mossy fibers extensively to most of the cerebellar cortex and nuclei (Wu et al., 1999). Although we do not constrain our principle to the cerebellum, this structure has been heavily implicated in timing production and perception (Ivry and Keele, 1989; Miall et al., 1993; Salman, 2002; Ivry and Spencer, 2004; Buhusi and Meck, 2005; Gooch et al., 2010). It is therefore plausible that LRN neurons could play the role of a population of clocks whose outputs are selectively monitored by downstream cerebellar circuitry.

The geometry and number of parallel fibers, the dendritic tree of cerebellar Purkinje cells and its feedforward inhibition by molecular layer interneurons are all ideally suited to the role of temporally precise coincidence detection (Braitenberg et al., 1997; Mittmann et al., 2005). An alternative site of convergence is the deep cerebellar nuclei, where patterns of input from the cerebellar cortex could be combined with timing signals from the LRN to drive appropriately timed responses. However, the general principle proposed by our model can be plausibly mapped onto numerous brain regions.

The number of convergent pacemaker neurons required in our model falls between the estimated total number of excitatory synapses onto a pyramidal neuron (Megias et al., 2001) and the estimated total number of parallel fiber synapses on the cerebellar Purkinje neuron (Napper and Harvey, 1988). We do not assume that the only function of each of this large number of inputs is to act as a pacemaker because such a large degree of redundancy could be costly and inefficient. We assume that only the coincidence detector neuron is dedicated for timekeeping. We envisage that timing can occur alongside other functions of the convergent neurons and they may send axonal collaterals to the dedicated timekeeping coincidence detector neuron. Under normal functions, these inputs would have insufficient synchrony to fire the coincidence detector. However, in response to certain stimuli sufficient numbers of the inputs may undergo a phase reset that enables them to act temporarily as pacemakers. Because this does not actually add or subtract any spikes in the post-reset period, other possible parallel downstream targets of the neurons might be unaffected if they normally undergo frequency encoding.

The post-synaptic coincidence detector's threshold in our model seeks to minimize both the mean error and the variability of responses. This is a strategy designed to compensate for the noisy nature of neurons; the existence of such a mechanism is conjecture. It would require a dynamic threshold that decreases when the response is too late and increases when the response is too early, as well as a strategy to avoid levels that give too great a temporal variability. This could arise out of a negative feedback of error, with a feedback gain changed according to response variability to avoid large oscillations in responses. However, what the biological underpinning of such a system might be is unclear.

### Synaptic plasticity

Previous experiments have demonstrated the existence of more than one shape of STDP function (Shouval et al., 2010). The function used in our model originally derives from *in vitro* results (Bi and Poo, 1998) which has since been adopted by numerous modeling studies (Song et al., 2000; Van Rossum et al., 2000; Gütig et al., 2003; Morrison et al., 2008). For our purposes the exact shape of the STDP function is not important as long as synapses generating coincident input at the right time are potentiated.

There is evidence that the amount of change in synaptic weight depends on the initial weight (Debanne et al., 1996, 1999; Bi and Poo, 1998). Therefore, we adopted a multiplicative weight update rule (Van Rossum et al., 2000; Gütig et al., 2003) which ensured that synaptic weights reached saturation more smoothly and produced a unimodal weight distribution after learning.

It has also not escaped our notice that spikes other than the adjacent pair around the post-synaptic spike can contribute to synaptic plasticity. However, given the relatively short and symmetrical time constant of our STDP function and the fact that in our model the pacemakers do not speed up or slow down their firing but simply reset after the cue, we have simplified things by modifying the “nearest-neighbor-takes-all” rule (Izhikevich and Desai, 2003) into an “adjacent-neighbors-take-all” rule.

### Scalar property and its violation

The scalar property refers to a proportionate relationship between the standard deviation of a response and the magnitude of the stimulus that drove it. Weber's law implies such a relationship and has been observed across a wide range of sensory modalities (Gibbon, 1977).

We propose a mechanism that underlies the temporal scalar property by explicitly taking into account the spike-wise accumulation of uncertainty in spike timing. Indeed such a mechanism could explain the observed phenomenon in associative learning where increasing the amount of time between the conditioning stimulus and the unconditioned stimulus decreases the speed of learning (Schneiderman and Gormezano, 1964). We also propose (and support with psychophysics data) that for short intervals the scalar property is violated because of both the linear fashion by which spike time variance accumulates and also the time-invariant source of variability in the time of the first post-reset spike.

### The possibility of different timing apparatus for different intervals

The general strategy proposed in our model could exist in more than one neuronal system. A clear result was that, due to the odd symmetry of the STDP function, the learned responses tended to be slightly early for short target times. It is conceivable that such a small bias could in reality be swamped by the additional variability introduced by variable subject attention and effector mechanisms to give the results seen for target times under 1 s in human subjects. For target times over 1 s human subjects showed a significant bias toward responding early and had a much greater degree of inter-subject variability in response variance. We show that the slight early bias of responses in our model can be increased if the mean inter-spike interval of the pacemaker population is made longer. However, this may require an anatomically distinct set of pacemakers. We suggest that in human subjects at least two different sets of time keeping apparatuses using the same general strategy could be responsible for encoding intervals below approximately 1 s and those over it. The apparatus encoding longer time intervals might even be more prone to differences in levels of attention and cognitive strategies between subjects. Our conjecture matches previous fMRI evidence suggesting that motor areas such as the cerebellum are used to measure sub-second intervals, whereas attentional regions such as the parietal lobes are used to measure supra-second intervals (Lewis and Miall, 2003).

## Author contributions

WX: design and implementation of model and psychophysics experiments. SB: design of model and psychophysics experiments.

### Conflict of interest statement

The authors declare that the research was conducted in the absence of any commercial or financial relationships that could be construed as a potential conflict of interest.
